# Use of ChemoFx® for Identification of Effective Treatments in Epithelial Ovarian Cancer

**DOI:** 10.1371/currents.eogt.8b0b6fffc7b999b34bc4c8152edbf237

**Published:** 2015-07-13

**Authors:** Scott Richard, Alan Wells, Joseph Connor, Fredric Price

**Affiliations:** Department of Obstetrics and Gynecology, Hahnemann University Hospital, Philadelphia, PA, USA; Department of Pathology, University of Pittsburgh; and Pittsburgh VA Health System, Pittsburgh, PA, USA; Department of Pathology and Laboratory Medicine, University of Wisconsin School of Medicine and Public Health, Madison, WI, USA; Department of Obstetrics and Gynecology, Allegheny Health Network, Pittsburgh, PA, USA

## Abstract

Selection of appropriate chemotherapy, including identification of platinum resistance, is critical to effective management of advanced epithelial ovarian cancer (EOC). ChemoFx®, a multiple treatment marker (chemoresponse assay), has been developed to address this challenge and to improve outcomes in patients with advanced EOC. While much work has been done that has demonstrated the analytical validity of this assay, more recent studies have highlighted the unique clinical benefits offered by the assay. A prospective, multicenter trial has shown an increase in overall survival (OS) of 14 months and an increase in progression-free survival (PFS) by 3 months in patients with recurrent EOS treated by a “sensitive” therapy based on ChemoFx results. Along with other studies showing similar gains in OS and PFS, ChemoFx has been shown to be both a prognostic and predictive marker in patients with recurrent EOC where current treatment options are sorely lacking. In addition to these clinical benefits, economic analyses have shown that ChemoFx is a cost-effective intervention. Current guidelines and technology assessments relating to ChemoFx are largely outdated and refer primarily to metrics of analytical validity. Thus, in addition to analytical validity, the clinical validity, clinical utility and economic impact of ChemoFx are reviewed herein, including published literature, technology assessments by independent parties, and regulatory approvals of this marker.

## Clinical Scenarios

Aggressive cytoreductive surgery followed by platinum-based chemotherapy is the standard first-line approach for the management of EOC. Currently, 6 different first-line chemotherapy regimens are recommended options in the National Comprehensive Cancer Network (NCCN) guidelines for Ovarian Cancer.^1^ Patient outcomes, however, are similar among the recommended options. Intravenous (IV) carboplatin/paclitaxel is the most commonly used regimen. While 60-80% of patients initially respond to this standard approach, the median PFS remains just 17 months and the median OS is 44 months.^2^ Therefore, in practically all women, the disease returns despite initial response to chemotherapy.

Selecting optimally effective treatment(s) for recurrent EOC is challenging. Considerations include: site(s) of recurrence, patient comorbidities, and cumulative toxicity from first-line chemotherapy. Repeat cytoreductive surgery and second-line chemotherapy are the most common treatments for recurrent or persistent disease. While most patients eventually succumb to EOC, many will experience prolonged remissions and symptom-free survival.^3^^,^ 4 Relapses in patients occurring more than 6 months from the completion of first-line therapy are considered to be ‘platinum-sensitive’. A portion of these patients will respond to re-exposure to platinum-based therapy. However, treatment guidelines outline numerous platinum-based regimens,^1^ with little or no difference in clinical performance for the population across the various options. Patients who progress during treatment or within 6 months of completion of first-line therapy are considered to be ‘platinum-resistant’, and decisions regarding second-line treatment are especially difficult. Platinum-resistant EOC is typically treated with sequential non-platinum single agent therapies. In general, the PFS and OS are poor for this group of patients compared to those who manifest platinum-sensitive disease. Currently, the treatment of EOC remains largely empiric. As with most clinical factors, the accuracy of platinum sensitivity and resistance is not absolute, and additional measures of responsiveness may be beneficial in personalizing treatment strategies.

## Test Description

As defined by the National Institutes of Health (NIH), a marker is “a characteristic that is objectively measured and evaluated as an indicator of normal biologic processes, pathogenic processes, or pharmacologic responses to a therapeutic intervention”.^5^ Therefore, ChemoFx is considered a marker of multiple treatments and provides tumor-specific information to assist physicians in the selection of effective chemotherapy for individual patients with gynecologic cancers. ChemoFx is intended to be utilized in conjunction with treatment guidelines, heuristics and pathways. Physicians indicate which of the multiple, guideline-recommended, FDA-approved chemotherapy options are under consideration for each patient when ordering the assay, and ChemoFx reports a result for each of the single agent or combination treatments selected for testing.

ChemoFx is a cell culture-based chemoresponse assay that measures the sensitivity of tumor-derived malignant epithelial cells to chemotherapeutic agents in vitro, using quantification of cellular DNA as the assay endpoint.^6^ The assay employs tumor tissue samples available at the time of clinically indicated surgery, biopsy or paracentesis; so, no additional procedures are required to fulfill tissue collection requirements for the assay. Tissue is shipped to the ChemoFx laboratory (Helomics™ Corporation, Pittsburgh, PA), and primary cultures are initiated by mincing each tissue sample into 1 mm^3^explants, which are then seeded into culture flasks. Upon near confluency, primary cultures are trypsinized and seeded into 384-well microtiter plates and used immediately for in vitro testing. Multiple concentrations of each treatment are prepared by serial dilution. Each concentration is added to three replicate wells on the microtiter plate; three replicates of control (no treatment) wells are also associated with each treatment. Culture seeding into microtiter plates, as well as serial treatment dilution and application, are completed using highly automated liquid handling robotics ensuring a consistent and repeatable process.^7^ After 72 hours of incubation with treatment, DNA in the nucleus of surviving adherent cells is stained with DAPI and counted using proprietary, automated, computer-assisted microscopy.^7^ The inhibition of tumor growth is measured for each concentration (average of cell counts in three replicates) of a given treatment. The survival fraction (SF) of tumor cells at each concentration is calculated as compared to control.^8^ The summation of SF values is computed as the drug response score, which represents the area under the dose response curve (AUC). A smaller AUC score indicates that a tumor is more sensitive to a treatment in vitro; a larger score indicates greater resistance to a treatment. For each treatment, in vitro tumor response is classified into one of three categories according to the AUC score: sensitive (S), intermediate sensitive (IS), or resistant (R). The cut-point threshold for the classifications were previously and independently established based on the 25th and 75th AUC percentiles in referent specimens, with an AUC score less than 25th rank classified as S, between 25th and 75th rank as IS, and greater than 75th rank as R.

*Unique, Distinguishing Features of ChemoFx*

ChemoFx is distinguished from prior chemoresponse assays in a number of ways:

- ChemoFx requires a small amount of cells or tissue (as little as 35 mm^3^, or smaller than the size of a pencil eraser), an amount that is typically easily accessible by surgery, biopsy or paracentesis.
- ChemoFx laboratory processes are highly automated. Robotic and computerized microscopy technologies allow for high throughput and exceptional reproducibility that is superior to other cell-based assays.
- The ChemoFx culture process is robust and preferentially supports outgrowth and proliferation of epithelial cells, using immunoctyochemisty to verify that the majority of the culture is made up of epithelial cells. Nearly 90% of specimens submitted for ChemoFx testing yield cultures that proceed through to the assay process.
- Cells cultured during the ChemoFx assay are actively cycling prior to chemotherapy exposure, ensuring that the efficacies of cell cycle-specific, cytostatic and cytotoxic agents are appropriately assessed.

*Genomic Relevance*

While ChemoFx is more a phenotypic, than a typical genomics assay, it represents the phenotypic response represents an integrated manifestation of genetic, genomic, proteomic and functional characteristics of a tumor. Published studies suggest that ChemoFx represents a novel platform for multigene (microarray) signature development, by associating in vitro assay response data with gene expression profiling data.^9^^,^ 10 ^,^ 11 Specifically, a multigene signature developed utilizing the ChemoFx platform has been independently validated, differentiating pathologic complete response from residual disease after neoadjuvant chemotherapy.^9^ The ability of ChemoFx to simultaneously assess response to multiple therapies makes this assay especially useful for developing genomic signatures that may predict differential response to therapies.

## Public Health Importance

It is estimated that, in 2015, there will be 21,290 new cases of ovarian cancer and 14,180 deaths due to this disease in the United States; EOC represents the leading cause of death from gynecologic cancer.^12^ The poor prognosis observed with EOC is largely attributed to detection of the disease at an advanced stage, as well as drug resistance at initial presentation or developed later. Although standard first-line treatment is initially effective for the majority of EOC patients, most of these patients will relapse within 1-2 years, and only 30% will live beyond 5 years.^13^Given this unfortunate prognosis, there is a strong desire, as well as clinical opportunity, to extend the overall survival of EOC patients. More than 30 different acceptable treatment choices are identified in current treatment guidelines for recurrent EOC.^1^ Yet, evidence on a population-wide level is insufficient to show that any one of the recommended regimens is superior to any others. ChemoFx can help guide personalized therapy decisions through the phenotypic approach of a chemoresponse assay. Further, in EOC, unlike some other solid tumors, biomarkers have not been well validated to document their ability to stratify patients for individualized treatment choices that improve outcomes. Recently published clinical trials provide evidence in support of the analytical validity, clinical validity and clinical utility of ChemoFx and are described in detail below. These new studies, including the first prospective clinical trials examining a chemoresponse assay, are published in the context of a significant of negative reviews of chemoresponse assays due to lack of prospectively designed clinical trials. These new studies address a need for validation proven through prospective clinical trials which has been requested for a number of years.

## Published Reviews, Recommendations and Guidelines

*Systemic evidence reviews/technology assessments*

The Blue Cross Blue Shield Association Technology Evaluation Center (BCBS TEC) evaluated chemoresponse assays in 1995 and subsequently issued minor updates in 2000 and 2002. BCBS TEC found that chemoresponse assays do not meet all of the TEC criteria, primarily due to a lack of prospective, randomized clinical trials to compare the outcomes of assay-guided treatment and empiric treatment.^14^^,^ 15 ^,^ 16 More than a decade has passed since the most recent BCBS TEC evaluation of 2002. Since then, multiple clinical trials, both retrospective and prospective, have reported on the clinical validity and clinical utility of ChemoFx,^17^^,^ 18 ^,^ 19 ^,^ 20 ^,^ 21 ^,^ 22 ^,^ 23 and, as such, these data were not included in any of the BCBS TEC assessments.

The American Society of Clinical Oncology (ASCO) also published technology assessments of chemoresponse assays in 2004 and 2011, with consideration of literature published through May 2010 for the 2011 assessment.^24^^,^ 25 Both ASCO assessments concluded that the then-current clinical literature did not support use of chemoresponse assays in routine oncology practice, citing low success rates, lack of appropriate prospective evaluation in clinical trials and a tendency to simply recommend treatments that would have been given empirically (i.e. low utility).^24^ However, the assessments noted the potential importance and impact of these assays and encouraged participation in clinical trials evaluating chemoresponse assays. It is, once again, noteworthy that the ASCO technology assessments were conducted prior to the publication of key clinical validations of ChemoFx which demonstrate the high technical success of ChemoFx compared to other CSRAs, prospective evaluation of the assay, and clinical utility by comparing clinical outcomes of patients with and without access to the assay.^18^^,^ 19 ^,^ 20 ^,^ 21 ^,^ 22 ^,^ 23 Specific details of these studies are noted below.

*Recommendations by independent groups*

The Helomics laboratory, where ChemoFx is performed, has undergone a number of evaluations of its analytical validity. The Wadsworth Center of the State of New York performed an independent review of the technology, standard operating procedures, quality measures, and analytical and clinical validation results of ChemoFx, resulting in approval and licensure in the state of New York. Furthermore, ChemoFx testing is performed in the Helomics Corporation laboratory in Pittsburgh, Pennsylvania, which is certified to comply with the Centers for Medicare & Medicaid Services Clinical Laboratory Improvement Amendments (CLIA) program. The ChemoFx laboratory is licensed by CLIA nationwide and also has specified licensure by the states of New York, California, Florida, Maryland, Pennsylvania and Rhode Island.

*Guidelines by professional groups*

In 2010, the NCCN Clinical Practice Guidelines in Oncology for Ovarian Cancer: Including Fallopian Tube Cancer and Primary Peritoneal Cancer were amended to state that “Chemosensitivity/resistance and/or other biomarker assays are being used in some NCCN Member Institutions for decisions related to future chemotherapy in situations where there are multiple equivalent chemotherapy options available.” Use of chemoresponse assays is classified as a category 3 level of evidence and has not been re-evaluated by the NCCN since 2010. ChemoFx has been used in 20 of 21 NCCN institutions. As with the above two situations the, this was completed before the most recent round of publications.

## Evidence Overview

*Analytical Validity*

- The reproducibility of ChemoFx has been evaluated in several studies and under numerous conditions.^7^^,^ 8 ^,^ 26 The coefficient of variance (CoV) in an ovarian cancer cell line, SK-OV-3, treated with doxorubicin has been measured to be 3.6-4.6% across three operators and over nine days.^7^ The CoV has been measured to be as low as 2-3% under conditions that mimic the current commercial process, which includes the use of liquid handling and process automation for plating cell cultures, preparing/diluting chemotherapy agents, treating cultures with chemotherapy agents, and fixing/staining cultures post-treatment, as well as counting live cells. Process variability due to the stability of chemotherapy agents (both within a given day and across multiple days) has also been measured and reported.^26^
- The primary cell culture process in ChemoFx enriches for malignant cells. In a study of 50 ChemoFx primary cultures, the percentage of malignant cells increased throughout the culture period for 86% (43/50) of them with an average magnitude of increase of 37%. Notably, the minimum proportion of malignant cells at the conclusion of the culture period across all 50 cultures was 60%.^6^

*Clinical Validity*

- A prospective, multicenter study demonstrated improved clinical outcomes (both PFS and OS) in patients with recurrent ovarian cancer who were treated with a therapy that was ‘sensitive’ according to the ChemoFx assay (PFS: HR=0.67, 95% CI=0.50-0.91, p=0.009; OS: HR=0.61, 95% CI=0.41-0.89, p=0.010).^19^ These improvements amounted to median increases in PFS of 3 months (9 vs. 6 months) and OS of 14 months (38 vs. 24 months). In multivariate analysis, ChemoFx result was shown to be independently associated with PFS (HR=0.66, 95% CI=0.47-0.94, p=0.020) and OS (HR=0.59, 95% CI=0.38-0.93, p=0.023). Improved clinical outcomes in patients treated with ChemoFx ‘sensitive’ therapies (as compared to patients treated with ‘non-sensitive’ therapies) were evident in both platinum-sensitive and platinum-resistant sub-populations. Furthermore, 52% of tumors demonstrated in vitro sensitivity to at least one agent, suggesting that, although generalized resistance is common in recurrent EOC, a majority of patients benefit from assay-informed treatment selections.
- Using the Rutherford, et al. cohort,^19^ four independent statistical methods were employed to assess the predictive properties of ChemoFx. All four analyses yielded the same result – ChemoFx has the ability to identify specific therapies that are likely to be more effective, predicting both response and prognosis. The association between ChemoFx result and clinical outcome was enhanced for the therapy used in clinical treatment (“match”), as compared to those that were randomly selected from all assayed therapies (“mismatch”) (PFS HR=0.67 vs. 0.81). Furthermore, improved outcome was associated with treatment with an assay-sensitive therapy, regardless of homogeneous (all sensitive or all resistant) or heterogeneous (mixed sensitive and resistant) responses among the assayed therapies.^21^
- Patients prospectively enrolled in an observational study and displaying in vitro resistance to carboplatin (via ChemoFx) had significantly shorter PFS after standard first-line therapy (carboplatin/paclitaxel) (HR=1.87, 95% CI=1.29-2.70, p<0.001), progressing 4.8 months sooner than patients displaying in vitro sensitivity to carboplatin (median PFS: 11.8 vs. 16.6 months). This association was confirmed in multivariate analysis to be independent of other relevant covariates (HR=1.71, 95% CI=1.12-2.62, p=0.013). Furthermore, for tumors that showed in vitro resistance to carboplatin, 59% displayed in vitro sensitivity to at least one non-platinum agent, suggesting that ChemoFx has the ability to narrow treatment choices in platinum-resistant disease. ChemoFx was shown to be independently associated with PFS in primary ovarian cancer patients. Patients predicted for poorer outcome (i.e. platinum resistance) by ChemoFx may be considered for alternate treatment options.^20^
- Primary ovarian cancer patients (n=192) treated with therapy categorized as sensitive via ChemoFx experienced a median OS more than twice as long (72.5 vs. 28.2 months) as patients treated with an assay-resistant treatment (HR= 0.70, 95% CI=0.504-0.968, p=0.031). ChemoFx prediction of response to platinum agents is an independent predictor of OS (HR=0.68, 95% CI=0.490-0.948, p=0.023).^18^
- In patients with evaluable disease, there was a statistically significant correlation between ChemoFx result and progression-free interval (PFI) in a retrospectively accrued, prospectively analyzed study of 135 EOC patients whose tumors were submitted for ChemoFx testing (HR=2.9, 95% CI=1.4-6.3, p<0.01). Patients treated with an assay-sensitive therapy experienced three-times longer PFIs compared to those treated with an assay-resistant therapy.^17^

*Clinical Utility and Other Supportive Studies*

- Clinical utility of ChemoFx was recently evaluated in an analysis of OS between advanced stage EOC patients (n=192) whose treatment was assay-informed and a combined cohort of >7000 primary EOC patients whose treatments were non-assay-informed. Despite a worse prognosis at baseline based on clinical covariates, assay-informed patients experienced a 10% improvement (48 vs. 44 months) in OS compared to non-assay-informed patients when not stratifying by an assay selected-treatment. When assay-informed patients were treated with an assay-sensitive therapy, they experienced a 28.5 month (65%) increase in OS (72.5 vs. 44 months), while those treated with an assay-resistant therapy demonstrated a 15.8 month (36%) decrease in OS (28.2 vs. 44 months).^22^
- In a survey of 23 gynecologic oncologists who have ordered a chemoresponse assay [conducted by an independent physician polling organization (Leerink Swann) in 2012], nearly all (95.7%) of the physicians indicated that assay results have helped them to decide between equivalent treatments. In addition, just over half (52.2%) of them maintained that assay results were used to select a less toxic and/or less expensive treatment.
- Metachronous paired tumors from 242 EOC patients exhibited in vitro chemoresponse profiles that, as clinically and biologically expected, displayed a general shift toward resistance over time. The shift towards increased resistance was more pronounced for therapies that are typically used in first-line treatment (carboplatin, cisplatin, paclitaxel, docetaxel) and, thus, are most likely to be previously administered. Collectively, the results of this study indicate that ChemoFx is most useful when a tumor sample is available immediately preceding a treatment decision; however, if a tumor sample is not obtained at recurrence, assay results obtained within the prior 17 months (median PFS for EOC after first-line treatment) may help select effective therapy, especially for therapies not previously administered.^27^

*Economic Impact*

- ChemoFx is considered to be a cost-effective health care intervention, using a Markov state transition model based on patient characteristics and survival data from a recent clinical study of ChemoFx in recurrent EOC [19]. ChemoFx presented an incremental cost effectiveness ratio (ICER) of $6,206 per life-year saved (LYS) for patients with recurrent EOC who are treated according to ChemoFx results (compared to similar patients whose treatment decisions were made without ChemoFx). Cost-effectiveness was further demonstrated in both platinum-sensitive and platinum-resistant populations treated with assay-sensitive therapies with ICERs of $2,773 per LYS and $2,736 per LYS, respectively. Furthermore, if the least expensive, sensitive therapy is chosen for treatment, use of ChemoFx has the potential to be cost saving.^23^
- The mean costs of chemotherapy treatment for recurrent ovarian cancer were estimated to be $48,758 for empirically treated patients, $33,187 for assay-assisted patients (oncologist’s choice of chemotherapy following chemoresponse testing, with 65% adherence to assay results) and $23,986 for assay-adherent patients (modeled group of patients assuming 100% adherence to assay results).^28^ According to this study, assay-assisted treatment decisions in recurrent ovarian cancer may result in reduced costs compared to empiric treatment selections.

## Limitations

Although a prospective, randomized controlled trial design has been recommended for use in the validation of markers due to its successful use in the validation of drugs, it is suggested that alternate study designs may be more appropriate for evaluating markers (especially tests that have the ability to report multiple markers simultaneously) which interface with and impact clinical scenarios differently than drugs.^29^^,^ 30 As such, clinical validations of ChemoFx employ a study design based on the marker-stratified (non-randomized, blinded) approach that has been successfully used in the validations of several markers that are considered to be standards of care in oncology (e.g. KRAS, Oncotype DX®, VeriStrat®).^31^^,^ 32 ^,^ 33 ^,^ 34 ^,^ 35 ^,^ 36 ^,^ 37 This approach overcomes the pragmatic obstacles of the design recommended in technology assessments (e.g. large sample size, heterogeneity of possible therapies) while allowing evaluation of multiple markers simultaneously and evaluating a marker’s predictive properties.

Although ChemoFx provides relative effectiveness within a given therapy (as compared to like patients), the comparative effectiveness between multiple therapies is not considered. Furthermore, other factors which influence treatment selection (e.g. toxicity) are not considered in the ChemoFx results, but remain as independent components of the patient’s profile that the physician considers when selecting the appropriate course of therapy.

## Conclusions

Outcomes for women with gynecologic cancers who have been empirically treated are stagnant and disappointing. ChemoFx helps clinicians individualize treatment selections and improve patient outcomes in gynecologic cancer. Numerous publications have outlined the strength of the analytical validity behind the ChemoFx assay in this setting. Several more recent studies, including those with a prospective design, have clinically validated that ChemoFx is an assay capable of predicting the effectiveness of specific therapies and that patients treated with ChemoFx sensitive therapies experience improved outcomes. Patients experienced improvements in OS of up to 14 months when treatments sensitive in ChemoFx were used clinically. Benefits of using ChemoFx, however, are not only limited to increases in OS and PFS. By limiting treatment with ineffective therapies, patients experience fewer unnecessary adverse effects and drug-related toxicities. These factors contribute to the evidence that has shown use of ChemoFx results in reduced overall treatment costs when compared to the current empiric based standard of care. The current evidence strongly suggests that ChemoFx provides a compelling and exciting option for improving the treatment paradigm and patient outcomes in EOC.

## Competing Interests

SR is a paid member of Helomics Corporation’s speakers bureau. AW is a paid consultant for Helomics Corporation and holds stock options with the company.
